# Effective dosage and mode of exercise for enhancing cognitive function in Alzheimer's disease and dementia: a systematic review and Bayesian Model-Based Network Meta-analysis of RCTs

**DOI:** 10.1186/s12877-024-05060-8

**Published:** 2024-06-01

**Authors:** Yuan Yuan, Yong Yang, XiaoFei Hu, Lin Zhang, Zhiyu Xiong, Ying Bai, JiaLe Zeng, Feng Xu

**Affiliations:** 1 Department of Physical Education, Kunsan National University, Daehak-RoJeollabuk-Do, Gunsan-Si, 541150 Korea; 2 Laboratory of Kinesiology and Rehabilitation, School of Physical Education and Sport, Chaohu University, Hefei, 238000 China; 3 The School of Physical Education, Handan University, Handan, 056005 China; 4 Department of Rehabilitation, West China Hospital Sichuan University Jintang Hospital, Chengdu, 610499 China; 5 The School of Physical Education and Health, East China Jiaotong University, Nanchang, 330013 China; 6 The School of Physical Education, Jiangxi Normal University, Nanchang, 330224 China; 7 College of Physical Education, Fujian Normal University, FuZhou, 350007 China

**Keywords:** Old adults, Alzheimer, Dementia, Exercise, Dose–response, RCTs, Network meta-analysis

## Abstract

**Objective:**

Research the dose–response relationship between overall and certain types of exercise and cognitive function in older adults with Alzheimer's disease and dementia.

**Design:**

Systemic and Bayesian Model-Based Network Meta-Analysis.

**Methods:**

In our study, we analyzed data from randomized controlled trials investigating the effects of different exercises on cognitive outcomes in older adults with AD. We searched the Web of Science, PubMed, Cochrane Central Register of Controlled Trials, and Embase up to November 2023. Using the Cochrane Risk of Bias tool (Rob2) for quality assessment and R software with the MBNMA package for data analysis, we determined standard mean differences (SMDs) and 95% confidence intervals (95%CrI) to evaluate exercise's impact on cognitive function in AD.

**Results:**

Twenty-seven studies with 2,242 AD patients revealed a nonlinear relationship between exercise and cognitive improvement in AD patients. We observed significant cognitive enhancements at an effective exercise dose of up to 1000 METs-min/week (SMDs: 0.535, SD: 0.269, 95% CrI: 0.023 to 1.092). The optimal dose was found to be 650 METs-min/week (SMDs: 0.691, SD: 0.169, 95% CrI: 0.373 to 1.039), with AE (Aerobic exercise) being particularly effective. For AE, the optimal cognitive enhancement dose was determined to be 660 METs-min/week (SMDs: 0.909, SD: 0.219, 95% CrI: 0.495 to 1.362).

**Conclusion:**

Nonlinear dose–response relationship between exercise and cognitive improvement in Alzheimer’s disease, with the optimal AE dose identified at 660 METs-min/week for enhancing cognitive function in AD.

**Supplementary Information:**

The online version contains supplementary material available at 10.1186/s12877-024-05060-8.

## Introduction

According to a recent report in 2023, the number of people with Alzheimer's in the United States alone has skyrocketed to 6.75 million and is projected to exceed 13.8 million by 2026 [1]. Alzheimer's dementia has become one of the prominent public health challenges of the twenty-first century [2]. At the same time, the enormous cost of Alzheimer's disease and dementia(AD) puts a huge strain on society and families, with studies showing that [3]: According to estimates made in 2019, the global annual societal cost of dementia was US^$^131.34 billion for 55.2 million people living with dementia. This equates to a cost of US^$^23,796 per person with dementia. Out of this amount, direct medical costs amounted to US^$^213.2 billion (16%), direct social sector costs (including long-term care) to US^$^448.7 billion (34%), and informal care costs to US^$^651.4 billion (50%). Moreover, in low- and middle-income countries, family caregivers of people with dementia face high levels of caregiving stress, adverse health effects of long-term care, and difficulties in managing the caregiving process. They also struggle to adapt to life changes and meet their own needs [4].

In the previous study, a comprehensive meta-analysis synthesizing findings from 43 prospective observational studies and 153 randomized controlled trials, Yu et al. established exercise as an effective intervention for preventing AD in older adults [5]. This conclusion is supported by similarities between animal models and human AD, including amyloid-beta deposition and tau protein pathology. Animal studies have further validated exercise's molecular basis for cognitive enhancement in AD, demonstrating its impact on reducing beta-amyloid deposition and improving cerebrovascular function [6, 7]. Additionally, a recent study by Holstein et al. in 'Nature Neuroscience' highlighted that exercise enhances brain health by increasing blood flow and promoting the circulation of cerebrospinal fluid [8]. Collectively, these findings underscore the role of regular exercise in boosting neurotrophic factor expression, exerting anti-inflammatory effects, and improving cognitive function and neuroplasticity.

However, different exercises have different effects on improving cognition in AD. Susana et. al. of the meta-analysis showed that aerobic exercise seems to significantly improve AD patients’ cognition [9]. Although studies have proven that aerobic exercise can improve the cognitive function of AD patients, the results of the network meta-analysis study by Shi et.al showed that resistance exercise was the most effective way to improve the cognitive function of AD patients [10]. In previous dose–response meta-analyses, the effects associated with resistance exercise on healthy older adults had yielded good insights [11, 12]. Additionally, the dose and response network meta of Daniel et. al. opened a new direction in the study of dose–response relationships and confirmed for the first time that the relationship between exercise dose and cognition in older adults was nonlinear and found older adults can achieve clinically meaningful benefits at doses lower than the WHO *(724 METs-min per week)* [13]. The varying effectiveness of exercise interventions on cognitive function in AD patients may largely be due to differences in exercise dosages. Previous dose–response studies often treated exercises within the same category as equivalent, regardless of their duration (e.g., equating a 40-min session with a 100-min session). Such an approach may overlook the distinct advantages of specific exercise types. For instance, it might ignore the benefits of strength training on bone density and metabolic rate or the cardiovascular benefits inherent to aerobic exercise [12, 14–16]. Moreover, the reliance on single-category dose–response modeling has made it challenging to accurately model the effects of different interventions. Additionally, while much of the existing research has focused on mild cognitive impairment a precursor to AD there has been a significant gap in studies specifically exploring the optimal exercise dosage and its impact on cognitive function in AD patients. Our study aims to fill this gap by providing nuanced insights into how different exercise doses can uniquely contribute to health outcomes in AD patients, representing a vital advancement in the field.

In order to fill the gap, our study follows the method used by Daniel et. al. to evaluate exercise intensity using task metabolic equivalents [13]. By utilizing a new dose–response model of network meta-analysis through a classical Bayesian prior theory model in probability [17, 18]. Our research meticulously evaluates existing randomized controlled trials on exercise interventions designed to enhance cognitive functions in AD. It delves into the nuanced, non-linear dynamics between the intensity and volume of exercise and the observed cognitive benefits. The study's core objective is to pinpoint the most effective exercise modalities for AD patients, alongside determining the optimal dosage for maximum cognitive improvement. This investigation is poised to substantially enrich evidence-based guidelines for exercise in managing cognitive symptoms of AD, thereby equipping healthcare professionals with robust data to inform their clinical decisions.

## Method

### Search strategy

This systematic review and network meta-analysis is registered on the international Prospective Register of Systematic Reviews site as CRD 42023484877, and it was reported following the PRISMA checklist [19]. We conducted a comprehensive literature search across Web of Science, PubMed, Cochrane Central Register of Controlled Trials, and Embase databases up to November 2023. To ensure the search was both thorough and precise, we crafted a strategy using medical subject headings *(MeSH)* and keyword searches specifically in PubMed, with three authors reviewing for accuracy and completeness. Our search utilized a combination of *MeSH* terms and synonyms including "Alzheimer”," "Dementia," "Aged," "Older adults," "Aging," "Cognitive impairment," and terms related to exercise such as "Physical Activity," "Exercise," "Training," "Resistance Exercise," and "Aerobic Exercise." We explicitly excluded studies on Mild Cognitive Impairment *(MCI)* using the term "NOT *(MCI, Mild Cognitive Impairment)*." Detailed search strategies, including the specific terms, dates, and methodologies employed, are documented in Appendix File 1*.*

### Study selection

We first imported the literature we retrieved into the Endnote 20 software (Clarivate Analytics, Philadelphia, PA, USA) to screen for duplicate articles. We also manually screened for duplicates. Secondly, we excluded animal experiments, conference abstracts, experimental protocols, guidelines, and reports, as well as non-English literature. Finally, we meticulously screened for and excluded reviews and meta-analyses to guarantee that our study exclusively incorporated RCTs. Title/abstract and full-text screening were conducted independently and in duplicate by two investigators *(Y.Y/X.F.H)*, with disagreements resolved by discussion or adjudication by a third author *(Yang. Y)*.

### Eligibility criteria

#### Types of participants

Participants must be diagnosed with AD and meet age criteria for older adults 65 years and older. Secondly, only studies focusing on cognitive impairment in healthy older adults and subjects clinically diagnosed with Mild cognitive impairment (MCI) were excluded. Furthermore, we also excluded studies of cognitive impairment due to other diseases (e.g. Parkinson's, stroke, diabetes, Attention Deficit/Hyperactivity Disorder, Epilepsy, Multiple Sclerosis, Autism, or Schizophrenia, etc.) and to ensure completely that the study looked at populations with AD.

#### Types of intervention

Previous research exercises had focused on increasing planned, structured activities. However, activities like gardening, daily tasks, household chores, and others with low task metabolic equivalents (METs, used to assess the intensity of exercise) do not result in muscle contraction sufficient to increase the body's calorie demand significantly. Therefore, it is challenging to make reasonable recommendations regarding appropriate exercise doses for AD [20].

In our study, both the intervention and control groups engaged in some form of physical activity. However, to be included in our intervention analysis, studies needed to specify the duration, frequency, and methods of exercise, quantifiable in *METs.* This research focused exclusively on the impact of exercise on cognitive impairment in AD, omitting studies that incorporated other interventions such as exercise combined with cognitive therapy, gardening, music therapy, or physiotherapy. This exclusion criterion was essential to isolate the cognitive benefits attributable solely to physical exercise. Moreover, the control group received standard care, including daily living guidance and health education, without additional exercise or specific health interventions. A comprehensive definition of the exercise interventions analyzed is available in Appendix File 9*.*

#### Types of outcome measures

In our studies, experiments that reported at least one outcome with one of the global cognitive measures were eligible for inclusion. (ex: MMSE [21], ADAS-Cog [22], MoCA [23]).

#### Types of studies

In order to keep the risk of bias at a low to moderate level, both published and unpublished randomized controlled trials (RCTs) were included in our study, whereas non-randomized controlled studies *(cohort studies, pathology-control, cross-sectional studies, *etc*.)* were excluded.

### Data extraction3

The data from studies that met the inclusion criteria *(Y.Y/X.F.H)* were extracted independently by two authors and disagreements were resolved by consensus among the third authors *(Yang. Y).* For each inclusion study, pertinent data and populated into an Excel spreadsheet. the researcher's name, published year of study, sample size (total/male/female), sex, age, intervention and control description, intervention period/frequency/minutes, cognitive assessment tool, and used transformation formulas for estimating the mean and standard deviation (Appendix file 2) any data that could be used to calculate effect size was extracted (Appendix file 3)*.* The formulas utilized for the calculation of Mean change and SD values were:

$${\text{Meanchange}}= \mathrm{ Meanpost}-{\text{Meanpre}}$$

$$SDchange=\sqrt{[({\text{SDpre}}2 +\mathrm{ SDpost}2) - (2 \times \mathrm{ Corr }\times \mathrm{ SDpre }\times \mathrm{ SDpost})]}$$

According to the guidelines of the Cochrane Handbook, the correlation coefficient (*Corr*) was set to 0.5 [24]. In addition to meeting the data analysis requirements of the Dose–Response network meta-analysis Package in R, we converted the standard errors (*SE*). n as sample size [25].

$$SE=\frac{SD}{\sqrt{n}}$$

In cases where the required data for dose–response meta-analyses could not be retrieved from published reports, we contacted the authors and requested additional data. In two studies, the authors were able to provide the required data after being contacted [26, 27].

### Data setting

First, the interventions were coded in two categories; Category I: the intervention and control groups were coded as "Exercise (PA)" and "Control (CON)". Exercise will be viewed as an overall equivalent (e.g., regardless of aerobic, anaerobic, exercise, etc.), with the aim of analyzing the optimal dose of overall exercise for AD patients. Category II: Interventions will be coded according to their primary form of PA: “Aerobic exercise” (AE), “Mixed exercise” (MIX), "Tai Chi” (TC), “Resistance training” (RT), “Exergame” (EX) and “control” (CON). The aim was to analyze the optimal dose and optimal modality of the different forms of exercise. At the same time, we chose metabolic equivalents of tasks *(METs)* to define exercise-specific energy expenditure [28]. Because METs provide a standardized way to quantify the intensity of different exercises. By measuring energy expenditure in terms of METs, the intensity of various sports and physical activities can be objectively compared, regardless of the type of activity or the individual performing the activity [29]. Not only, by calculating METs-min consumed per week, our study took into account not only the duration and frequency of exercise *(METs-min/week* = *duration minute* × *times-pre week* × *MET value)* but also the intensity of exercise, which is critical to assess its impact on health outcomes [20, 30, 31]. Additionally, in order to facilitate the connectivity required for Network Meta-analysis, the intervention intensity was classified into five different groups with weekly controls set at 0,250, 500, 750,1000, and 1250 METs-min and it’s proved in the previous study [32, 33].

### Data synthesis

All data analysis was performed in R version 4.0.3 [34]. We used the *"MBNMAdose"* package to analyze the reticulation dose–response relationship which exercise dose and AD cognition impairment [17, 35]. We used the Emax functional model, restricted cubic spline, non-parametric model, exponential model different fitting metrics for random and fixed effects models to select the best-fitting model for analyzing our study data. such as DIC *(deviation information criterion)*, standard deviation, parameters in the model, and residuals [36]. With the results (Appendix File 5 Table 2)*,* the restricted cubic spline model of the random effects model was found to have a better fit. Therefore, in our study, we opted restricted cubic spline of the random effects model [18, 37, 38]. In addition, to visualize the best functional model fit to our data, we plotted relative line and box plots of the deviation of equivalent exercises from different exercises in Appendix File 5.

At the same time, we checked the data for three key hypotheses of network meta-analysis network connectivity, consistency (Appendix Table 1)*.*, and transitivity (Appendix 4: Figure, 4) [37, 39, 40]. Additionally, because included studies assessed cognitive function using different measurement scales, effect measures were pooled as standardized mean differences *(SMDs),* SMDs do not rely on the specific units of the original scale and therefore can be used to combine results from studies that use different measurement units and ranges, eliminating interpretation barriers that may arise when using raw score differences directly, meanwhile with 95% credible intervals (95% CrI) to assess the credibility of our estimates [41, 42].

In order to estimate the overall and different exercise doses that resulted in the predicted maximum significant effect referred to as the ‘optimal exercise dose’. We summarized the result of the dose–response relationship by the MCMC model (Markov Chain Monte Carlo Iterations) [3 chains, 20,000 iterations each (first 10,000 discarded), n. thin = 10] of the beta coefficients on the restricted cubic spline curves by “rjags” package in R [43]. We positioned the three nodes at the 10th, 50th, and 90th percentile of the exercise dose to visualize our model-fitting results. The code to reproduce the results presented in this paper can be accessed at the first author's e-mail address.

### Risk of bias and quality of evidence

Our study was selected according to the Cochrane (Rob2) criteria [44–46]. and packages “robvis” was used to plot the results in R. Three reviewers (Y.Y/X.F.H/Yang. Y) assessed the study, we assessed only five categories of risk of bias, including randomized sequence generation, bias due to deviation from the intended intervention, incomplete data, bias in measurements, and selective bias in reporting results. Disagreements were resolved by the third author. Additionally, we performed sensitivity analyses and excluded high-risk bias studies to determine the robustness of the overall exercise dose–response model [47] (Appendix File 8)*.*

## Results

### Description of included studies

**Fig. 1:**
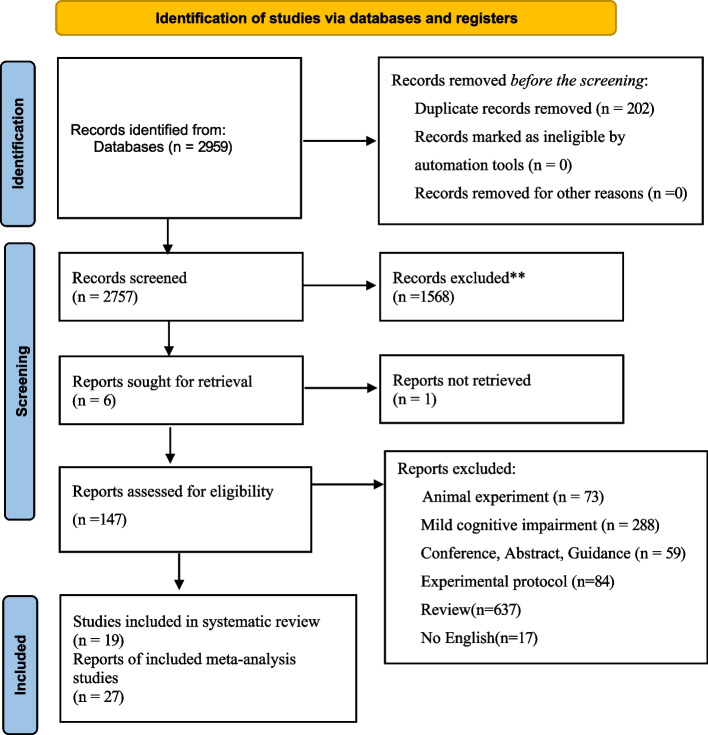
PRISMA Flow diagram of the search process for studies. *RCT* randomized controlled trials

A total of 1962 potentially eligible studies were searched. After removing literature that did not fit my study by title, abstract, etc., we considered 147 studies that were potentially eligible for inclusion and retrieved full-text articles. After deleting duplicates and applying the inclusion criteria, there were 27 RCT studies [26, 27, 48–72] included in this analysis were published between 2006 and 2023 (Fig. 1)*.* Out of the total of 2242 participants, 1210 (54%) were male and 1032 (46%) were female. All patients included in the studies had AD and were aged between 65 and 85 years old. In these studies, the exercise interventions in the 17 studies have AE, 8 studies have Mixed, 3 studies have RT, 3 studies have Taiichi and 2 studies were game-based exercise interventions and we provided Characteristics information for the included study in Table 1. More information about the characteristics of the included studies can be found in Appendix File 3*.*

**Table 1:** Characteristics of the included studies

Characteristics	% (No) of studies (*N* = 27)
**Mean age of participants**	
65–69.9	3 (0.11)
70–74.9	4 (0.15)
75–79.9	15 (0.56)
80–84.9	5 (0.19)
**Sample Size**	
10–19	11 (0.41)
20–29	4 (0.15)
30–50	9 (0.33)
> 50	3 (0.11)
**Intervention**	
Aerobic Exercise dosage	AE 250 Mets-min/week: 4 (0.15) AE 500 Mets-min/week: 7 (0.26) AE 750 Mets-min/week: 4 (0.15) AE 1200 Mets-min/week: 1 (0.04)
Exergame exercise dosage	EG 250 Mets-min/week: 1 (0.04) EG 1200 Mets-min/week: 1 (0.04)
Mix Training dosage	MIX 500 Mets-min/week: 5 (0.19) MIX 750 Mets-min/week: 1 (0.04) MIX 1000 Mets-min/week: 2 (0.07)
Resistance Training dosage	RT 250 Mets-min/week: 1 (0.04) RT 750 Mets-min/week: 2 (0.07)
Taichi dosage	TC 250 Mets-min/week: 2 (0.07) TC 750 Mets-min/week: 1 (0.04)
**Exercise period (weeks)**	
< 12	4 (0.15)
12–23	9 (0.33)
24–48	14 (0.52)
**Exercise Frequency**	
1 times/week	1 (0.04)
2 times/week	7 (0.26)
3 times/week	13 (0.48)
> 3 times/week	6 (0.22)
**Session Duration (min)**	
10–30 min	11 (0.41)
31–60 min	14 (0.52)
> 60 min	2 (0.08)
**WHO Recommendations**	
< 600 METs*min/week	19 (0.70)
600 METs*min/week—1200 METs*min/week	8 (0.30)
**Outcome**	
MMSE	20 (0.74)
MoCA	2 (0.07)
ADAS-Cog	5 (0.19)

### Network connectivity

**Fig. 2:**
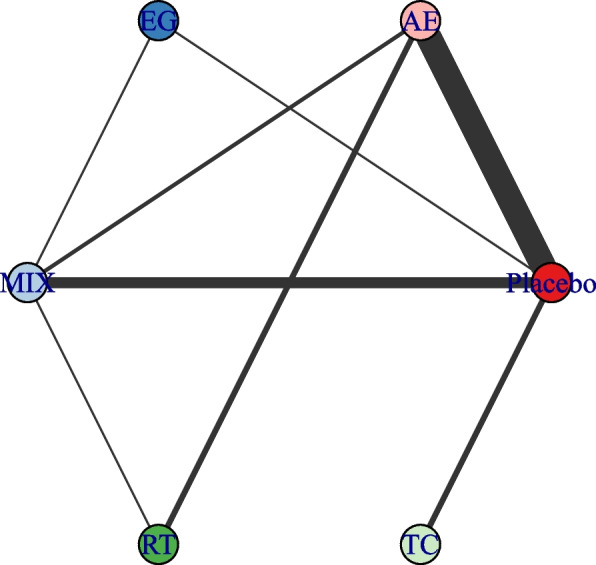
Treatment level. The first value indicates the specific intervention AE Aerobic Exercise, CON Control group, MT Multicomponent Exercise Program, RT Resistance Training, TC: Taichi, EX Exergame exercise

**Fig. 3:**
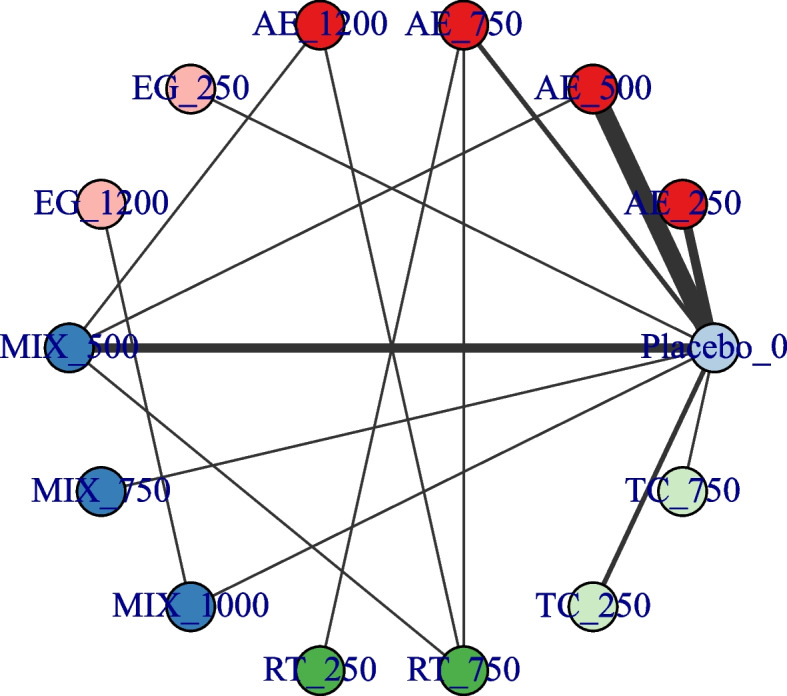
Agent-level network plot. the value indicates the corresponding dose of that intervention. AE Aerobic Exercise, CON Control group, MT Multicomponent Exercise Program, RT Resistance Training, TC: Taichi, EX Exergame exercise

Whether connectivity is met determines the basis of NMA. Lack of connectivity can lead to low statistical power and misleading results when direct comparison is not possible [73]. The analysis confirmed no connectivity deficit in the two networks, ensuring the accuracy of the results (Figs. 2 and 3).

### Dose–response relationship

**Fig. 4:**
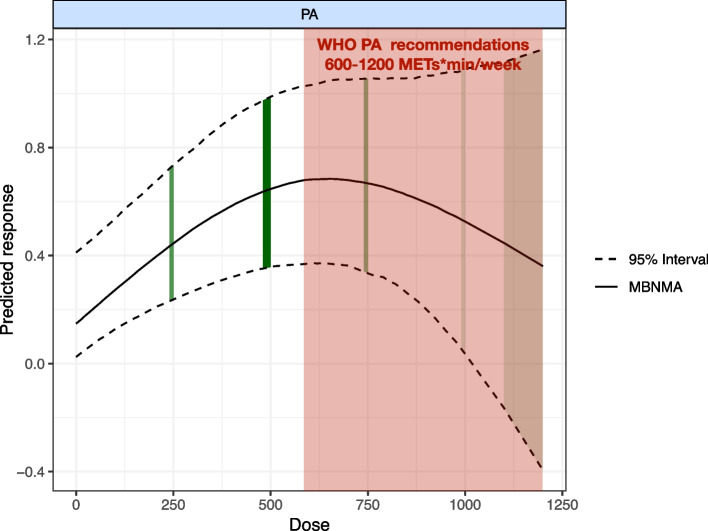
Dose–response association between overall exercise dose and change in cognitive function in AD, the exercise dose distribution is represented by the green part in our study. The red part indicates the WHO-recommended exercise dose range. PA overall exercise

Figure 4 shows there was a nonlinear dose–response relationship between overall exercise dose and cognition up to 1000 METs -min/week (SMDs: 0.535, SD: 0.269, 95%Crl: 0.023 to 1.092), with overall exercise showing a significant increase in cognitive function. Above 1000 METs-min/week, the response to cognitive function was significantly diminished. Meanwhile, the optimal dose of overall exercise was estimated at 650 METs-min/week (SMDs:0.691, SD:0.169, 95%Crl: 0.373 to 1.039) for improving cognitive function with AD.

**Fig. 5:**
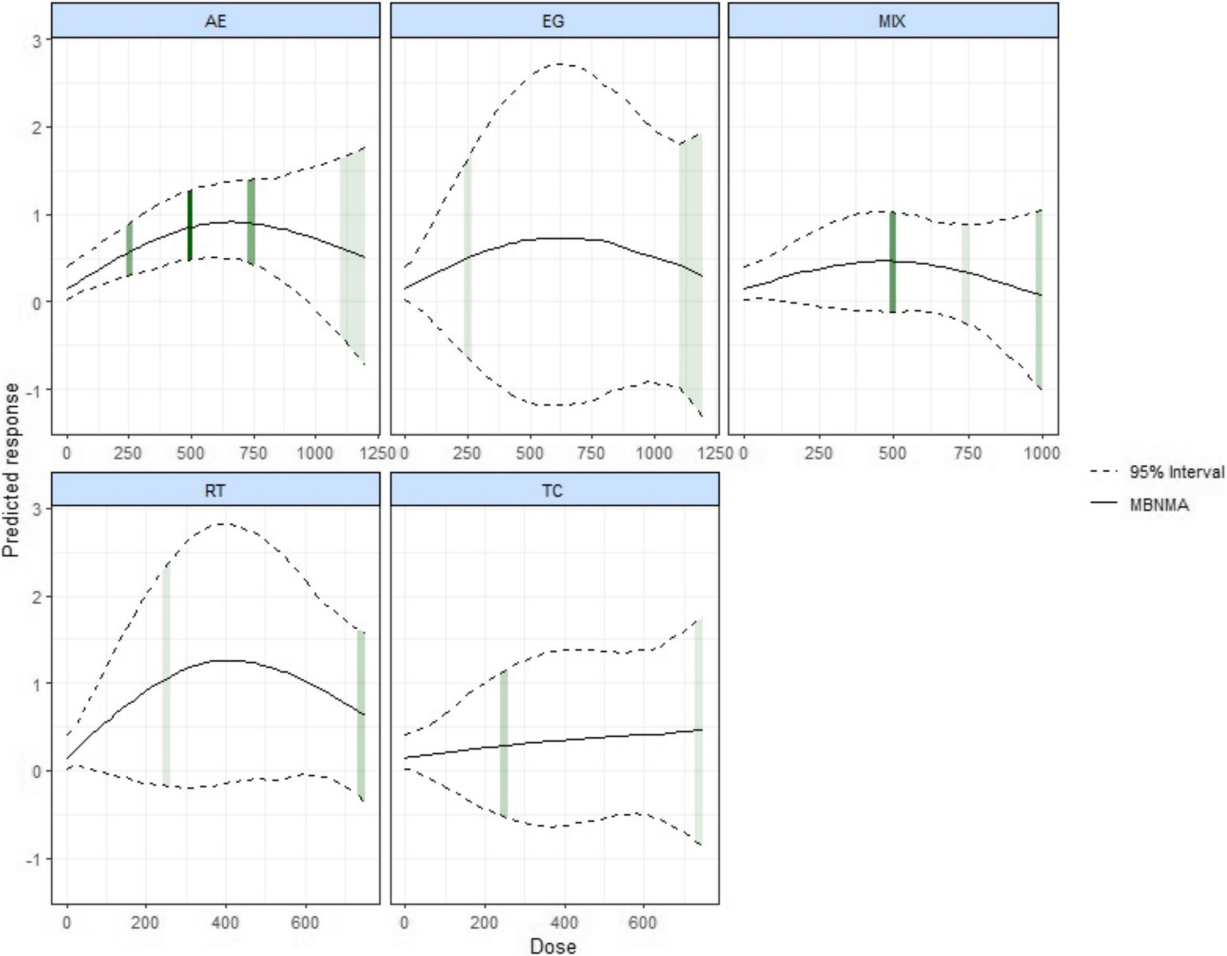
Dose–response association between different exercise doses and change in cognitive function in AD. AE Aerobic Exercise, CON Control group, MT Multicomponent Exercise Program, RT Resistance Training, TC: Taichi, EX Exergame exercise

In Fig. 5, we show the dose–response curves for different types of exercise. Surprisingly, AE, EG, and MIX of maximum dose exceed 1000METs-min/week, however, EG did not appear to be effective in improving cognitive function in AD within the dose range. At the same time, we found an inverted U-shaped relationship between exercise dose and cognition function for AE. The optimal AE dose was found at 660 METs -min/week (SMDs: 0.909, SD:0.219, 95% CrI: 0.495 to 1.362). The improvement of cognition effect was not significant for AE at over 980 METs-min/week (SMDs: 0.729, SD: 0.374, 95%CrI: 0.004 to 1.501). On the other hand, mixed exercise was estimated to be effective at improving cognitive function at a small dose level until 180 METs-min/week (SMDs: 0.324, SD: 0.171, 95%Crl: 0.004 to 0.683). It is worth exploring that RT and TC have not found any dose to improve cognitive function in AD.

### Risk of bias and certainty of evidence

**Fig. 6:**
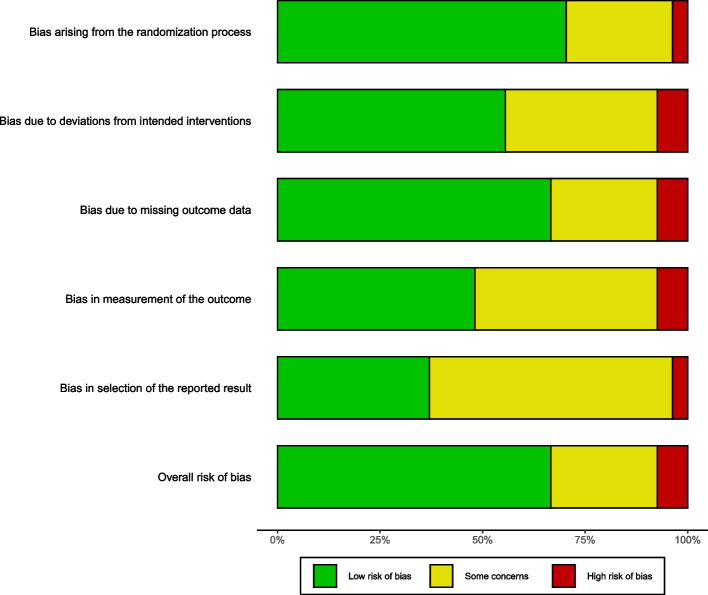
Cochrane Risk of Tool

Thirteen studies had a low risk of bias, 11 studies had a moderate risk of bias, and 4 studies had a high risk of bias (Fig. 6). Study-level risk of bias assessments are presented in Appendix File 7. Sensitivity analyses that included only studies with a low risk of bias were consistent with the results of the main analysis (Appendix File 7). The overall quality of the evidence was moderate according to the GRADE system. After excluding studies at high risk of bias (Appendix File 8). The optimal dose of the overall exercise was estimated at 760METs-min/week (SMDs: 0.7663, SD: 0.298, 95%CrI: 0.205 to 1.392) in improving cognitive function. The significance of improving cognitive deficits in AD was not significant at above 880METs-week (SMDs: 0.753, SD: 0.3653, 95%CrI: 0.0535 to 1.526).

## Discussion

### Main findings

Our study is the first to explore the nonlinear relationship between exercise dosage and cognitive function in AD, revealing that exercise positively influences cognitive impairment in AD patients. This systematic review and network meta-analysis encompassed 27 randomized controlled trials, involving 2,242 AD patients. Our findings indicate that aerobic, mixed exercise significantly enhances cognitive functions in AD patients. and that the optimal dose of overall exercise to improve cognitive dysfunction in elderly patients with AD was 650 METs-min/week. This also corresponds to approximately 150 min of moderate-intensity exercise per week or 75 min of vigorous exercises per week. This result could provide a theoretical basis for future pairs of clinical trials and indirectly demonstrate the clinical feasibility implications of our study [74]. In the analysis of the results of different exercise modalities to improve cognitive deficits in AD patients, it was concluded that 660 METs -min/week of aerobic exercise was the optimal dose to improve cognitive function in AD, which is consistent with the results of previous Susana et. al. study [9]. Our study expands upon the research conducted by Susana et al., offering a detailed exploration into the optimal dosage of specific exercise intensities for enhancing cognitive function in AD patients. This investigation delves further into the empirical validity of the findings, aiming to refine and substantiate the recommended exercise protocols for AD patients.

### Strengths

There are several key strengths to our study. First, our study's use of metabolic equivalents of task (METs) to assess exercise intensity lies in its simplicity, versatility, and ability to easily compare different activities. METs are relative values of resting metabolic rate, allowing use by people across age, gender, and culture, facilitating the formulation of public health guidelines and public understanding. In addition, METs can quantitatively compare various activity intensities, supporting individuals and health professionals in developing and adjusting exercise plans [28]. Second, our study included a relatively large sample size of AD, and in order to achieve the study aim, our study provided adequate statistical power. Third, we applied the current state of the newest meta-analytical techniques to investigate the dose–response between exercise and the improvement of cognition function in AD. The new method allowed us to determine the effective dose of exercise and the optimal dose of different modalities for cognitive improvement in AD patients. Also, order to WHO recommendations, exercise improves cognitive function in patients with AD, and indirectly proves any level of exercise is better than no exercise [74, 75]. Our research results not only determined that exercise within the effective dose range is 1000 METs-min/week but also identified 650 METs-min/week as the optimal dose to improve cognitive function in AD. The actual recommendations of the WHO (600 ~ 1200METs-min/week) are echoed, and we have confirmed the effectiveness of the WHO through the existing evidence and helped medical staff better understand the strength of the WHO recommendations. Fourth, our study utilizes direct, indirect, and network estimation methods to compare the relative efficacy of various exercise interventions. This comprehensive approach enabled us to identify exercise as the most effective intervention for enhancing cognitive function in AD. We found that all types of exercise evaluated are associated with improvements in overall cognition. However, aerobic exercise has a more significant interaction with overall cognition than other types of exercise. Comparison to previous studies reporting that resistance exercise improves overall cognition in dementia or MCI populations [13, 76], The results of our study indicated that only aerobic exercise was effective in improving cognitive function in patients with AD. This was due to the strict control of inclusion and exclusion criteria, which focused specifically on AD. On the other hand, the study found a weaker dose response of mixed exercise with resistance training to improve cognitive function in patients with AD. This may be the reason why there are too few randomized controlled trials for relevant AD and more randomized controlled trials for AD patients are expected in the future, seeking to prove the effectiveness of mixed exercise versus resistance exercise on cognition in AD.

### Limitations

There are several limitations in our study. Firstly, we were unable to conduct a more in-depth statistical analysis of heterogeneity due to the small number of included studies. Instead, we used a risk assessment tool to assess bias. Secondly, we did not thoroughly analyze some potential covariates such as education level, gender, and weight of the elderly, which could contribute to heterogeneity in the study results. Thirdly, we categorized the methods of evaluating cognitive impairment in AD patients into global cognitive impairment evaluation (primarily using scale evaluation) and executive cognitive impairment evaluation methods (assessing working memory, switching, and inhibition). Our primary outcome focused on the global cognitive impairment scale evaluation. Although our study identifying optimal exercise doses for AD patients provides an evidence-based recommendation aimed at promoting cognitive health, its applicability may vary due to global cognitive impairment and executive cognition. Functional impairment varies with different types of cognitive impairment. Individual differences, such as the patient's performance level, disease stage, and specific type of cognitive impairment, all need to be considered to ensure the effectiveness and safety of the exercise program. Therefore, although 650 METs-min/week provides a useful starting point, individualized adjustment of exercise dose and a combination of multimodal interventions may better meet the individual needs of AD patients, thereby maximizing positive effects on cognition and function. Fourth, our study only included English language literature, potentially leading to missing data from researchers in other countries and limiting the generalizability of our results.

Lastly, although metabolic equivalents of exercise (METs) are a widely used metric for assessing exercise intensity, which quantifies the intensity of different exercises in terms of their ratio relative to resting-state energy expenditure, in practice, the application of METs faces several significant limitations [77]. First, the calculation of METs is based on the average resting metabolic rate, without considering individual differences that affect energy expenditure, such as age, gender, weight, and physical condition. This means that the same activity may represent different actual intensities for different individuals. Second, MET values provide a fixed estimate of energy expenditure for an activity but lack the sensitivity to capture subtle changes in activity intensity, especially when distinguishing between high- and low-intensity exercise. In addition, the resting metabolic rate of all individuals is assumed to be a uniform standard (1 MET), ignoring the actual differences in energy expenditure in the resting state between people. METs are also difficult to accurately assess complex exercises or contain multiple levels of intensity, and to accurately measure exercise intensity in everyday settings where specialized measurement equipment is not available. Finally, standard MET values do not apply to individuals with specific health conditions because it does not reflect the unique responses of these individuals to exercises. Therefore, while METs provide a convenient metric for rapid estimation of exercise intensity, to obtain a more accurate and personalized assessment, other methods including heart rate monitoring are recommended, taking into account the individual's specific health status and energy expenditure characteristics.

### Clinical implications and directions for future research

Our study not only corroborates previous findings on the efficacy of aerobic exercise in enhancing cognitive function in AD patients but also found a specific dose–response relationship, identifying an optimal aerobic exercise dose of 660 METs-min/week [9]. Furthermore, we established that the overall exercise dose aligns with the World Health Organization's recommended range. By pinpointing effective modalities and dosages for cognitive improvement in AD patients, our research paves the way for future exercise guidelines. In addition, implementing exercise interventions for AD patients presents significant logistical challenges. Effective execution of aerobic exercise programs demands skilled supervision and considerable resources. Comprehensive planning is essential, incorporating policy development, economic considerations, and future research directions [78]. Both government bodies and the private sector must invest in public health policies and infrastructure. This investment should focus on creating safe and accessible exercise venues, alongside professional and public education initiatives to heighten awareness of AD and the advantages of regular exercise. Concurrently, conducting cost-effectiveness analyses can highlight the potential of exercise interventions to reduce the long-term financial burden of AD care. Additionally, financial policies and incentives aimed at promoting investments in exercise programs for the prevention and management of AD are crucial. This multifaceted approach is key to enhancing the feasibility and success of exercise interventions in the AD patient population, ultimately contributing to improved health outcomes and reduced societal costs.

## Conclusion

Our study incorporates the latest Bayesian modeling 'MBNMAdose' package to determine the dose–response relationship between different types of exercise and cognitive function in patients with AD. We found that the optimal overall exercise and AE dose. Using these findings, scientifically prescribed exercise can help us better cope with the cognitive function of AD by developing appropriate exercise prescription guidelines. Furthermore, given the limitations of the previously explained meta-analyses and the insufficient number of studies in the existing literature, it is important to interpret the results with caution. In the future, more detailed randomized controlled trials using a randomized group approach with different exercise doses are recommended to obtain more direct evidence on the relative effectiveness of exercise dose and response in different exercise interventions. In addition, the baseline physical tolerances of different AD patients should be fully considered to develop a rational exercise prescription programmer.

### Supplementary Information

Additional file 1: Appendix 1. Search Strategy. Appendix 2. Transformation formulas for estimating the mean and standard deviation. Appendix 3. Characteristics of the dataset and included studies. Appendix 4. Key Assumptions of Network Meta-Analysis. Appendix 5. Nonlinear function and model fit comparison. Appendix 6. Ranking of the effectiveness of interventions. Appendix 7. Study level Risk of Bias analysis. Appendix 8. Sensitivity analysis including only studies with low risk of bias. Appendix 9. The list of included studies.

## Data Availability

If reviewers want to repeat the results of our study data, Please contact the first author by e-mail.

## Abbreviations

RCT: Randomized controlled trials
AD: Alzheimer's disease and dementia
PA: Exercise
SMD: Standardized Mean Difference
SD: Standard Deviation
95%Crl: 95% Credible Interval
AE: Aerobic exercise
MIX: Mixed exercise
RT: Resistance training
TC: Tai chi
EG: Exergame exercise
CON: Control group
