# Supplementary material for: Effective dosage and mode of exercise for enhancing cognitive function in Alzheimer's disease and dementia: a systematic review and Bayesian Model-Based Network Meta-analysis of RCTs
Source: BMC Geriatr. 2024 Jun 1;24:480. doi: 10.1186/s12877-024-05060-8 (PMC11143595; doi:10.1186/s12877-024-05060-8)
Supplement: Supplementary file 1 — Additional file 1: Appendix 1. Search Strategy. Appendix 2. Transformation formulas for estimating the mean and standard deviation. Appendix 3. Characteristics of the dataset and included studies. Appendix 4. Key Assumptions of Network Meta-Analysis. Appendix 5. Nonlinear function and model fit comparison. Appendix 6. Ranking of the effectiveness of interventions. Appendix 7. Study level Risk of Bias analysis. Appendix 8. Sensitivity analysis including only studies with low risk of bias. Appendix 9. The list of included studies. [file 12877_2024_5060_MOESM1_ESM.docx]

**Effective Dosage and Mode of Physical Activity for Enhancing Cognitive Function in Alzheimer's Disease and Dementia: A Systematic Review and Bayesian Model Based on Network Meta-analysis of RCTs**

[1.Appendix File 1: Search strategy 1](#_Toc161340939)

[2.Appendix File 2: Transformation formulas for estimating the mean and standard deviation 8](#_Toc161340940)

[3.Appendix File 3: characteristics of dataset and included studies 10](#_Toc161340941)

[4.Appendix File 4: Key assumption of network meta-analysis 20](#_Toc161340942)

[*Consistency* 20](#_Toc161340943)

[Appendix Table 1. Consistent and UME models fit comparison 20](#_Toc161340944)

[Appendix Figure 1. Validation Model Consistency Scat te rplot 20](file:////Users/yoyocandance/Desktop/副本Supplementary%20file%201.docx#_Toc161340945)

[*Transitivity* 21](#_Toc161340946)

[Appendix Table. 2 comparison of transitivity 21](#_Toc161340947)

[Appendix Figure 2. Node splitting analysis (density plot) 23](file:////Users/yoyocandance/Desktop/副本Supplementary%20file%201.docx#_Toc161340948)

[5.Appendix File 5: Nonlinear functions and models fit comparison 24](#_Toc161340949)

[Appendix Figure 3. “Split” NMA of overall exercise 24](#_Toc161340950)

[Appendix Figure 4. “Split” NMA of different PA agents 25](#_Toc161340951)

[Appendix Table 3. Models fit comparison 26](#_Toc161340952)

[Appendix Figure 5. Deviance plot at overall PA level 27](file:////Users/yoyocandance/Desktop/副本Supplementary%20file%201.docx#_Toc161340953)

[Appendix Figure 6. Deviance plots at PA level 28](file:////Users/yoyocandance/Desktop/副本Supplementary%20file%201.docx#_Toc161340954)

[6.Appendix File 6:studylevel Risk of Bias analysis 29](#_Toc161340955)

[Appendix Figure 7. Studylevel Risk of Bias analysis 29](#_Toc161340956)

[7.Appendix File 7: Sensitivity analysis including only studies with low 30](#_Toc161340957)

[Appendix Figure 8. Doseresponse curve between exercise and changes in cognitive function after including only studies with low risk of bias 30](#_Toc161340958)

[8.Appendix File 8: Definitions of exercise types and non-exercise training control 31](#_Toc161340959)

[9.Appendix File 9: The list of included studies 32](#_Toc161340960)

1.[Appendix File 1: Search strategy](#_heading=h.17dp8vu)

| 1.1 Web of science  （2023.08.18） | 1.Exercise.tw  2.Activity.tw  3.Physical.tw  4.Physical activity.tw  5.Resistance.tw  6.Strength.tw  7.Resistance exercise.tw  8.Strength exercise.tw  9.Aerobic exercise.tw  10.Taichi.tw  11.Taiji.tw  12.Baduan jin.tw  13.Yoga.tw  14.Qigong.tw  15.Dance.tw  16.Boxing.tw  17.RunninAg.tw  18.Walking.tw  19.Training.tw  20.Plyometric.tw  21 .#1 OR #2 OR #3 OR #4 OR #5 OR #6 OR #7 OR #8 OR #9 OR #10 OR #11 OR #12 OR #13 OR #14 OR #15 OR #16 OR #17 OR #18 OR #19 OR #20 **(12184114)**  22.aged.tw  23.elder.tw  24.senior.tw  25.”old people”.tw  26.”old abult”.tw  27.”againg”.tw  28. #22 OR #23 OR #24 OR #25 OR #26 OR #27**(5215524)**  29.Alzheimer Dementia.tw  30.Alzheimer Dementias.tw  31.Dementia, Alzheimer.tw  32.Alzheimer's Disease.tw  33.Dementia, Senile.tw  34.Senile Dementia.tw  35. Dementia, Alzheimer Type.tw  36.Alzheimer Type Dementia.tw  37.AlzheimerType Dementia (ATD).tw  35.Alzheimer Type Dementia (ATD).tw  36.Dementia, Alzheimer Type (ATD).tw  37.Alzheimer Type Senile Dementia.tw  38.Alzheimer.tw  39.Dementia.tw  40.#29 OR #30 OR #31 OR #32 OR #33 OR #33 OR #34 OR #35 OR #36 OR #37 OR #38 OR #39**(359247)**  41.cognitive.impairment.tw  42.cognition disorders.tw  43.congnition.tw  44.cognitive dysfunction  45.mild cognitive impairment.tw  46.MCI  47. #41 OR #42 OR #43 OR #44 NOT #45 NOT #46**(353486)**  48.control trial.tw  49.RCT.tw  50.Randomized controlled trial.tw  51.randomized controlled trial.tw  52.#48 OR #49 OR #50 OR #51(813906)  53. #21 AND #28 AND #40 AND #47 AND #52**(1962)** |
| --- | --- |
| 1.2 Pubmed  （2023.08.18） | Exercise[MEsH Terms]**（249717）**  1.exercise[Title/Abstract]  2.Activity[Title/Abstract]  3.Physical[Title/Abstract]  4.Physical activity[Title/Abstract]  5.Resistance[Title/Abstract]  6.Strength[Title/Abstract]  7.Resistance exercise[Title/Abstract]  8.Strength exercise[Title/Abstract]  9.Aerobic exercise[Title/Abstract]  10.Taichi[Title/Abstract]  11.Taiji[Title/Abstract]  12.Baduan jin[Title/Abstract]  13.Yoga[Title/Abstract]  14.Qigong[Title/Abstract]  15.Dance[Title/Abstract]  16.Boxing[Title/Abstract]  17.RunninAg[Title/Abstract]  18.Walking[Title/Abstract]  19.Training[Title/Abstract]  20.Plyometric[Title/Abstract]  21.Exercise([MEsH Terms]) OR #1 OR #2 OR #3 OR #4 OR #5 OR #6 OR #7 OR #8 OR #9 OR #10 OR #11 OR #12 OR #13 OR #14 OR #15 OR #16 OR #17 OR #18 OR #19 OR #20**（5620543）**  22.aged[Title/Abstract]  23.elder[Title/Abstract]  24.senior[Title/Abstract]  25.”old people”[Title/Abstract]  26.”old abult”[Title/Abstract]  27.”againg”[Title/Abstract]  28. #22 OR #23 OR #24 OR #25 OR #26 OR #27**（797454）**  29.”Alzheimer Disease”[MeSH]**(120739)**  30. "Dementia"[Mesh]**(208111)**  31.cognitive.impairment[Title/Abstract]  32.cognition disorders[Title/Abstract]  33.congnition[Title/Abstract]  34.cognitive dysfunction  35.mild cognitive impairment[Title/Abstract]  36.MCI[Title/Abstract]  37. #30 OR #31 OR #32 OR #33 NOT #34 NOT #35**（168400）**  38. exercise（MeSH）AND #21 AND #28 AND #30 AND #29 AND #36**(488)** |
| 1.3 Cochrane  （2023.08.18） | 1.exercice (MeSH)**(38702)**  2. Activity  3.Physical  4.Physical activity  5.Resistance  6.Strength  7.Resistance exercise  8.Strength exercise  9.Aerobic exercise  10.Taichi  11.Taiji  12.Baduan jin  13.Yoga  14.Qigong  15.Dance  16.Boxing  17.RunninAg  18.Walking  19.Training  20.Plyometric  21.#1 OR #2 OR #3 OR #4 OR #5 OR #6 OR #7 OR #8 OR #9 OR #10 OR #11 OR #12 OR #13 OR #14 OR #15 OR #16 OR #17 OR #18 OR #19 OR #20**(646564)**  22.aged  23.elder  24.senior  25.old people  26.old abult  27.againg  28. #22 OR #23 OR #24 OR #25 OR #26 OR #27(892516)  29.Alzheimer disease (MsSH)**(5391)**  30.Dementia (MsSH)**(9936)**  31.#29 OR #30**(9336)**  32. cognitive.impairment  33.cognition disorders  34.congnition  35.cognitive dysfunction  36.mild cognitive impairment  37.MCI  38.#32 OR #33 OR #34 OR #35 NOT #36 mild cognitive impairment NOT #37 MCI **(103967)**  39.control trial  40.RCT  41.Randomized controlled trial  42.randomized controlled trial  43.#39 OR #40 OR #41 OR #42**(902655)**  44.#21 AND #28 AND #31 AND #38 AND #43(202) |
| 1.4 Embase  （2023.08.18） | 1.'exercise'/exp**(449304)**  2.Activity :ab.ti.kw  3.Physical :ab.ti.kw  4.Physical activity :ab.ti.kw  5.Resistance :ab.ti.kw  6.Strength :ab.ti.kw  7.Resistance exercise :ab.ti.kw  8.Strength exercise :ab.ti.kw  9.Aerobic exercise :ab.ti.kw  10.Taichi :ab.ti.kw  11.Taiji :ab.ti.kw  12.Baduan jin :ab.ti.kw  13.Yoga :ab.ti.kw  14.Qigong :ab.ti.kw  15.Dance :ab.ti.kw  16.Boxing :ab.ti.kw  17.Running :ab.ti.kw  18.Walking :ab.ti.kw  19.Training :ab.ti.kw  20.Plyometric :ab.ti.kw  21.#1 OR #2 OR #3 OR #4 OR #5 OR #6 OR #7 OR #8 OR #9 OR #10 OR #11 OR #12 OR #13 OR #14 OR #15 OR #16 OR #17 OR #18 OR #19 OR #20**(7149047)**  22.aged:ab.ti.kw  23.elder:ab.ti.kw  24.senior:ab.ti.kw  25.old people:ab.ti.kw  26.old abult:ab.ti.kw  27.againg:ab.ti.kw  28. #22 OR #23 OR #24 OR #25 OR #26 OR #27**(1100967)**  29.`Alzheimer disease`/exp**(250100)**  30.’dementia’/exp**(450938)**  31.#29 OR #30**(450938)**  32.cognitive impairment:ab.ti.kw  33.cognition disorders :ab.ti.kw  34.congnition:ab.ti.kw  35.cognitive dysfunction:ab.ti.kw  36.mild cognitive impairment:ab.ti.kw  37.MCI:ab.ti.kw  38.#30 OR #31 OR #32 OR #33 NOT mild cognitive impairment NOT MCI**(239888)**  39.control trial:ab.ti.kw  40.RCT:ab.ti.kw  41.Randomized controlled trial:ab.ti.kw  42.randomized controlled trial:ab.ti.kw  43.#37 OR #38 OR #39 OR #40 **(206489)**  44.#21 AND #28 AND #31 AND #38 AND #43**(307)** |

**2.Appendix File 2: Transformation formulas for estimating the mean and standard deviation**

(1) When calculating SD from M (confidence interval) for intervention or control group.

| A.The sample size ＞100 |
| --- |
| 1. SE= (upper limit to lower limit)/3.92 2. SD=SE*N^ (1/2) |

| B.The sample size in each group≤100 |
| --- |
| 1. Input “tinv (10.95, N11)” in Microsoft Excel to obtain t 2. SE= (upper limit to lower limit)/ t 3. SD=SE*N^(1/2) |

(2) When calculating SD from MD and P value between intervention or control group

| A. reporting the exact p value |
| --- |
| 1. Input “tinv (p, N1+N22)” in Microsoft Excel to obtain t 2. SE=MD/t 3. SD=SE/N1^(1/2) |

(3)

| When calculating M (SD) from the m (interquartile range) for intervention or control group |
| --- |
| 1. the sample size＞25, M=m; 2. the sample size≤25, M=(𝑎+2m+𝑏)/4 3. the sample size≤15, SD=(((𝑎−2𝑚+𝑏)2/4+(𝑏−𝑎)2)/12) ^ (1/2) 4. the sample size 15<n≤70, SD=(𝑏−𝑎)/4 5. the sample size＞70, SD=(𝑏−𝑎)/6 |

(4)

| When calculating M (SD) from the m (interquartile range) for intervention or control group |
| --- |
| 1. M= (𝑞1 +𝑚+𝑞3)/3  2. calculation of SD  Z=(0.25𝑁1+0.375)/(𝑁1+0.25)  Input “norm. inv(1z,0,1)” obtain μ  SD=(𝑞3−𝑞1)/(2∗𝜇) |

***Note: M= mean; SE=standard error of mean; SD=standard deviation; N=sample size; MD= mean difference; m=median; a= minimum value; b = maximum value; q1= first quartile; q3= third quartile.***

**3.Appendix File 3: characteristics of dataset and included studies**

***This appendix file reveal the datasets used in this study.***

1. The *studyID* indicates the name of the author and the year of publication.
2. The *sample* indicates the number of participant.
3. The *age* indicates the average age of intervention and control.
4. The *sex* indicates the number of males and females.
5. The *y* indicates the effect size (Mean_change_).
6. The *se* indicates the standard error of the mean.
7. The *intervention* indicates the type of protocol style.
8. The *METs_session* indicates the energy expenditure in a session of the study.
9. The *Protocol pameters* of indicates the frequency of days and duration minute those participants were involved in physical activity.
10. The *exact_dose* parameter indicates the exact estimated METs per week that participants accumulated in the study.
11. The *dose* indicates the group of doses by approximation.
12. The *residual_dose* indicates the difference between the exact dose and the dose allocated by approximation.
13. The *outcome* indicates the evaluation tool used from study.

| **StudyID** | **sample** | **age** | **sex** | **y** | **se** | **intervention** | **Mets_session** | **Protocol**  **parameters** | **exact_dose** | **dose** | **Residual_dose** | **outcome** |
| --- | --- | --- | --- | --- | --- | --- | --- | --- | --- | --- | --- | --- |
| Nicola et al. 2008 | 69 | 68.6 ±8.7 | Males32/ Female37 | 1.6 | 0.317681821585813 | Aerobic exercise | 3.5 | Session duration:24weeks  Frequency:3 times  Total duration:50min | 525 | 500 | 25 | ADASCog |
| Nicola et al. 2008 | 69 | 68.7 ±8.5 | Males33/Female36 | 1.7 | 0.28303736136357 | Usual care | 0 | Session duration:24  weeks  Frequency:3 times  Total duration:50min | 0 | 0 | 0 | ADASCog |
| Kwak et al. 2008 | 15 | 79.67 ± 6.64 | NA | 4.54 | 1.555341338 | Aerobic exercise | 3.5 | Session duration:48  weeks  Frequency:3 times  Total duration:35min | 367.5 | 250 | 117.5 | MMSE |
| Kwak et al. 2008 | 15 | 82.27 ± 7.09 | NA | 1.2 | 1.773072664 | Usual care | 0 | Session duration:48weeks  Frequency:3 times  Total duration:35min | 0 | 0 | 0 | MMSE |
| Gilles et al. 2010 | 16 | 82.0±5.8 | 4/12 | 3.57 | 1.7803581 | Aerobic exercise | 3.5 | Session duration:15  weeks  Frequency:3 times  Total duration:60min | 630 | 750 | 120 | ERFC |
| Gilles et al. 2010 | 15 | 81.7±5.1 | 4/11 | 5.1 | 2.018222001 | Usual care | 0 | Session duration:15weeks  Frequency:3 times  Total duration:60  min | 0 | 0 | 0 | ERFC |
| Tai et al. 2011 | 14 | 70.21±7.90 | 9/5 | 1.15 | 1.748221556 | Taichi | 2.2 | Session duration:6  weeks  Frequency:2  times  Total duration:180min | 792 | 750 | 42 | MMSE |
| Tai et al. 2011 | 10 | 76.3±7.07 | 5/5 | 0.5 | 1.460749813 | Usual care | 0 | Session duration:6weeks  Frequency:2times  Total duration:180min | 0 | 0 | 0 | MMSE |
| Holthoff et al. 2012 | 15 | 72.44±4.34 | 7/8 | 0.06 | 0.555607775 | Aerobic exercise | 3.5 | Session duration:12  weeks  Frequency:3 times  Total duration:30  min | 315 | 250 | 65 | MMSE |
| Holthoff et al. 2012 | 15 | 70.67±5.41 | 8/7 | 1.23 | 0.545068804 | Usual care | 0 | Session duration12weeks  Frequency:3 times  Total duration:30min | 0 | 0 | 0 | MMSE |
| Bossers et.al 2015 | 37 | 85.7±5.1 | 8/29 | 1.35 | 0.709394486 | Mixed exercise | 3.9 | Session duration12weeks  Frequency:3 times  Total duration:30min | 468 | 500 | 32 | MMSE |
| Bossers et.al 2015 | 36 | 85.4 ±5.4 | 8/28 | 0.28 | 0.8646017 | Aerobic exercise | 3.5 | Session duration12  weeks  Frequency:3 times  Total duration:30min | 420 | 500 | 80 | MMSE |
| Bossers et.al 2015 | 36 | 85.4 ±5.0 | 11/25 | 0.72 | 0.724461483 | Usual care | 0 | Session duration12weeks  Frequency:3 times  Total duration:30min | 0 | 0 | 0 | MMSE |
| Telenius et.al 2015 | 82 | 86.9 ±7 | 23/59 | 0.1 | 0.581734164 | Mixed exercise | 4 | Session duration12weeks  Frequency:3 times  Total duration:30min | 440 | 500 | 60 | MMSE |
| Telenius et.al 2015 | 81 | 86.4 ±7.8 | 20/61 | 0.6 | 0.579058411 | Usual care | 0 | Session duration9  weeks  Frequency:4  times  Total duration:30  min | 0 | 0 | 0 | MMSE |
| José et.al 2016 | 116 | 80.63 ± 8.32 | 41/32 | 1.5 | 0.50675832 | Aerobic exercise | 3.5 | Session duration9  weeks  Frequency:4 times  Total duration:30  min | 420 | 500 | 80 | MMSE |
| José et.al 2016 | 73 | 82.90 ± 7.42 | 22/94 | 0.46 | 0.69310606 | Usual care | 0 | Session duration9  weeks  Frequency4  times  Total duration:30  min | 0 | 0 | 0 | MMSE |
| yang et.al 2015 | 25 | 72.00±6.69 | 10/15 | 0.05 | 0.215351319 | Aerobic exercise | 3.5 | Session duration60weeks  Frequency5times  Total duration:15min | 262.5 | 250 | 12.5 | MMSE |
| yang et.al 2015 | 25 | 71.92±7.28 | 7/18 | 0.19 | 0.575566211 | Usual care | 0 | Session duration60weeks  Frequency5 times  Total duration:15min | 0 | 0 | 0 | MMSE |
| Kristine et.al 2016 | 93 | 69.8 ±7.4 | 56/51 | 0.1 | 0.336650165 | Aerobic exercise | 6.8 | Session duration12weeks  Frequency3 times  Total duration:30min | 612 | 500 | 112 | MMSE |
| Kristine et.al 2016 | 107 | 71.3 ±7.3 | 57/36 | 0.2 | 0.410515197 | Usual care | 0 | Session duration  12 weeks  Frequency  3 times  Total duration:30min | 0 | 0 | 0 | MMSE |
| Annika et.al 2017 | 93 | 84.4±6.2 | 23/70 | 1.15 | 0.42608451 | Mixed exercise | 4 | Session duration16weeks  Frequency3times  Total duration:45min | 540 | 500 | 40 | MMSE |
| Annika et.al 2017 | 93 | 85.9±7.8 | 22/71 | 0.93 | 0.42327204 | Usual care | 0 | Session duration16  weeks  Frequency3  times  Total duration:45min | 0 | 0 | 0 | MMSE |
| Sarah et.al 2018 | 137 | 78.1±7.7 | 86/51 | 0.2 | 0.57279449 | Mixed exercise | 6 | Session duration48weeks  Frequency2times  Total duration:75min | 900 | 1000 | 100 | MMSE |
| Sarah et.al 2018 | 278 | 76.9±9.7 | 166/112 | 0.4 | 0.662168527 | Usual care | 0 | Session duration48  weeks  Frequency2  times  Total duration:75min | 0 | 0 | 0 | MMSE |
| Massimo et.al 2018 | 12 | 83 ±6 | NA | 1 | 0.603022689 | Aerobic exercise | 3.5 | Session duration24weeks  Frequency4times  Total duration:30min | 420 | 500 | 80 | MMSE |
| Massimo et.al 2018 | 12 | 85 ± 5 | NA | 6 | 0.632455532 | Usual care | 0 | Session duration124weeks  Frequency4times  Total duration:30min | 0 | 0 | 0 | MMSE |
| Cynthia et.al 2018 | 10 | 78.5 (64 81.2) | 4/6 | 0.3 | 0.758946638 | Aerobic exercise | 3.5 | Session duration16weeks  Frequency2times  Total duration:30min | 210 | 250 | 40 | MMSE |
| Cynthia et.al 2018 | 10 | 79 (74.7 82.2) | 5/5 | 2.1 | 0.252982213 | Usual care | 0 | Session duration16weeks  Frequency2times  Total duration:30min | 0 | 0 | 0 | MMSE |
| Julian et al. 2019 | 11 | 63.92 ±5.19 | 6/6 | 0 | 0.187737727 | Aerobic exercise | 3.5 | Session duration26weeks  Frequency3times  Total duration:50min | 525 | 500 | 25 | MMSE |
| Julian et al. 2019 | 12 | 65.88 ±4.00 | 6/5 | 0.59 | 0.312263242 | Usual care | 0 | Session duration26weeks  Frequency3times  Total duration:50min | 0 | 0 | 0 | MMSE |
| Huang et al. 2019 | 40 | 81.9 ± 6.0 | 17/28 | 0.45 | 1.014770083 | Taichi | 2.2 | Session duration40weeks  Frequency3times  Total duration:20min | 132 | 250 | 118 | MMSE |
| Huang et al. 2019 | 40 | 81.9 ± 6.1 | 14/26 | 1.32 | 0.886918075 | Usual care | 0 | Session duration40weeks  Frequency3times  Total duration:20min | 0 | 0 | 0 | MMSE |
| Samuel et al. 2019 | 42 | 77.9±8.3 | 24/18 | 1.7 | 0.966235533 | Taichi | 2.2 | Session duration20weeks  Frequency1times  Total duration:45min | 99 | 250 | 151 | MMSE |
| Samuel et al. 2019 | 43 | 78.2±7.5 | 27/16 | 1.4 | 0.942489098 | Usual care | 0 | Session duration：20weeks  Frequency：1 times  Total duration:45 min | 0 | 0 | 0 | MMSE |
| Arnaldina et al. 2019 | 19 | 84.8 ± 5.9 | 4 /15 | 0.4 | 1.376494403 | Mixed exercise | 3.5 | Session duration24  weeks  Frequency2  times  Total duration:50 min | 350 | 250 | 100 | MoCA |
| Arnaldina et al. 2019 | 18 | 83.3 ± 5.3 | 5 /13 | 2.8 | 0.967241207 | Usual care | 0 | Session duration24  weeks  Frequency2  times  Total duration:50min | 0 | 0 | 0 | MoCA |
| Liu et 73al. 203920 | 30 | 86.77±6.99 | 24/6 | 1.5 | 0.840470113 | resistance training | 5 | Session duration：4  weeks  Frequency5  times  Total duration:30min | 750 | 750 | 0 | MMSE |
| Liu et al. 2020 | 31 | 84.68±6.74 | 16/15 | 2 | 0.746780185 | Aerobic exercise | 4.8 | Session duration4weeks  Frequency5times  Total duration:30min | 720 | 750 | 30 | MMSE |
| Joeke et al. 2020 | 73 | 79.0 ±6.0 | 37/36 | 0.2 | 0.821542149 | Exgame exercise | 3.8 | Session duration24weeks  Frequency2  times  Total duration:30min | 228 | 250 | 22 | MMSE |
| Joeke et al. 2020 | 39 | 79.0±7.0 | 23/16 | 2.4 | 0.996275115 | Usual care | 0 | Session duration24weeks  Frequency2times  Total duration:30min | 0 | 0 | 0 | MMSE |
| Liao et al. 2021 | 25 | 79.6 ±9.0 | 9/16 | 2.7 | 1.09672238 | Exgame exercise | 7.2 | Session duration12 weeks  Frequency6 times  Total duration:60min | 1296 | 1200 | 96 | MoCA |
| Liao et al. 2021 | 21 | 83.8 ±5.1 | 6/15 | 1.3 | 1.333273798 | Mixed exercise | 5.3 | Session duration12weeks  Frequency13times  Total duration:60min | 954 | 1000 | 46 | MoCA |
| Yu et al. 2021 | 53 | 77.0 ± 6.6 | 77±6.6 | 1 | 0.856826091 | Aerobic exercise | 6.8 | Session duration24 weeks  Frequency3 times  Total duration:35min | 714 | 750 | 36 | ADASCog |
| Yu et al. 2021 | 25 | 78.9 ± 5.6 | 78.9±5.6 | 0.1 | 1.06226174 | resistance training | 2.3 | Session duration24 weeks  Frequency3 times  Total duration:35min | 241.5 | 250 | 8.5 | ADASCog |
| Yu et al. 2021 | 64 | 77.4 ± 6.6 | 36/28 | 2.4 | 4.80475025 | Aerobic exercise | 6.8 | Session duration24weeks  Frequency3times  Total duration:35min | 714 | 750 | 36 | ADASCog |
| Yu et al. 2021 | 32 | 77.5 ± 7.1 | 17/15 | 2.2 | 5.22047532 | Stretch | 2.3 | Session duration24  weeks  Frequency3  times  Total duration:35  min | 241.5 | 250 | 8.5 | ADASCog |
| Flavia et al. 2022 | 18 | 74.33 ±5.87 | 5/13 | 1.6 | 3.709999988 | Mixed exercise | 5.3 | Session duration24  weeks  Frequency2  times  Total duration:60  min | 636 | 750 | 114 | ADASCog |
| Flavia et al. 2022 | 18 | 81.83 ±6.18 | 6/12 | 0.3 | 3.35 | Usual care | 0 | Session duration24  weeks  Frequency2  times  Total duration:60  min | 0 | 0 | 0 | ADASCog |
| Holthoff et al. 2012 | 15 | 72.44±4.34 | 7/8 | 1 | 1.118033989 | Aerobic exercise | 3.5 | Session duration16weeks  Frequency4times  Total duration:30min | 420 | 500 | 80 | MMSE |
| Holthoff et al. 2012 | 15 | 70.67±5.41 | 8/7 | 2 | 1.612916618 | Usual care | 0 | Session duration16  weeks  Frequency：4  times  Total duration:30min | 0 | 0 | 0 | MMSE |
| Kim et al. 2023 | 19 | 81.9±7.0 | 6/13 | 0.13 | 1.041158533 | Aerobic exercise | 3.5 | Session duration：24  weeks  Frequency：5 times  Total duration:60  min | 420 | 500 | 80 | ADASCog |
| Kim et al. 2023 | 14 | 80.9±6.1 | 2/12 | -2.18 | 1.417828789 | Usual care | 0 | Session duration：24  weeks  Frequency：5 times  Total duration:60  min | 0 | 0 | 0 | ADASCog |
| Rami et al.  2023 | 20 | 78.89 ±6.68 | 10/10 | 0.5 | 0.720690635 | Aerobic exercise | 6.8 | Session duration：24  weeks  Frequency：2 times  Total duration:40  min | 1125 | 1200 | 75 | MMSE |
| Rami et al.  2023 | 20 | 78.92 ± 8.04 | 10/10 | 0.3 | 0.686178069 | resistance training | 4.2 | Session duration：24  weeks  Frequency：2 times  Total duration:40  min | 693 | 750 | 57 | MMSE |
| Rami et al.  2023 | 20 | 78.4 ± 6.21 | 10/10 | 0.05 | 0.841091558 | Mixed exercise | 3.6 | Session duration：24  weeks  Frequency：2 times  Total duration:40  min | 426 | 500 | 74 | MMSE |

**4.Appendix File 4: Key assumption of network meta-analysis**

There are three key assumptions to conducting a Network Meta-Analysis (NMA):

(1) consistency in the data

(2) transitivity in the data

***Consistency***

We carried out consistency analysis in the data through the comparison of consistent (i.e., network effect sizes) and unrelated mean effects (UME) models (i.e., pairwise effect sizes) of the network.

In practice, we checked whether deviance, the number of estimated parameters in the network, and the Deviance Informative Criterion (DIC) indicators were similar for both models which would indicate a good fit. Comparison of these parameters indicated good consistency across models (Appendix Table 1)

***Appendix Table 1.*** ***Consistent and UME models fit comparison***

| **Model** | **pD** | **Residual deviance** | **DIC** | **SD** |
| --- | --- | --- | --- | --- |
| **Consistent** | **51** | **56.387** | **189.5** | **1.648** |
| **UME** | **47.7** | **56.206** | **185.5** | **1.390** |

*Note. pD: Number of estimated parameters; DIC: Deviance Informative Criterion; SD: Standard*

*Deviation; UME: Unrelated Mean Effects. Scientific literature indicated that the main indicator to*

***
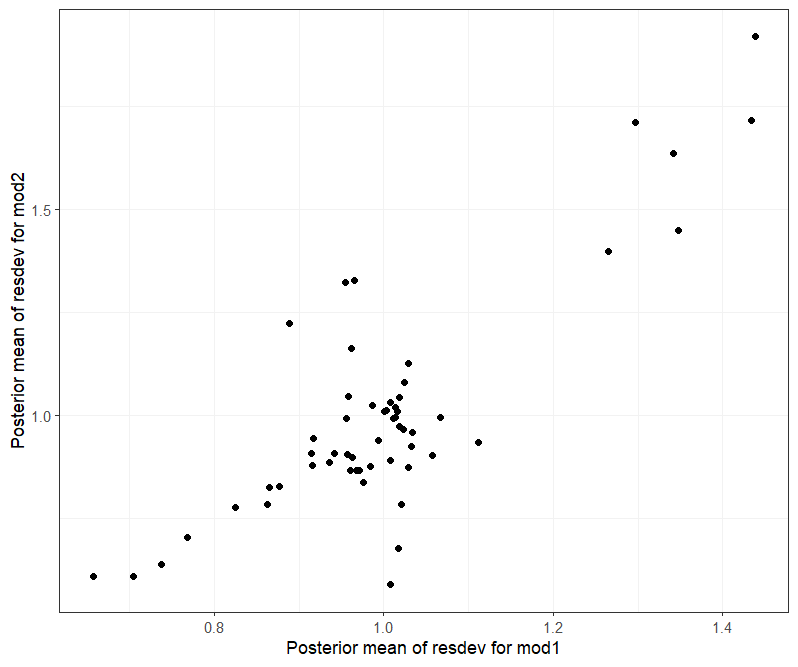
****assess the model fit is the DIC. As lower DIC, better fit.*

*Appendix Figure 1. Validation Model Consistency Scat te rplot*

***Transitivity***

NMAs rest under the assumption of underlying indirect/mixed comparisons, which means the estimates of treatment effects from direct and indirect evidence are in agreement, subject to the usual variation under the random effects model for meta-analysis. This assumption is equivalent to heterogeneity in ‘standard’ meta-analysis. Following previous recommendations, transitivity was assessed at the deeper level of the network (i.e., treatment level). We assessed transitivity MBNMA node splitting approach. This method splits and compares contributions for a particular treatment contrast into direct and indirect evidence. Similar effects denote good transitivity. Appendix Table 2 and Figure.4 (density plots) below present the results for transitivity in this meta-analysis.

***Appendix Table. 2 comparison of transitivity***

| Comparison p-value Median 2.5% 97.5%  MIX_1000 vs EG_1200 0.900  -> direct -0.233 -1.321 0.842  -> indirect -0.133 -1.474 1.219  -> MBNMA -0.191 -1.028 0.595  RT_750 vs AE_1200 0.853  -> direct -0.084 -1.076 0.888  -> indirect 0.049 -0.753 0.839  -> MBNMA -0.058 -0.673 0.537  RT_250 vs AE_750 0.577  -> direct 0.159 -0.911 1.165  -> indirect -0.229 -0.813 0.368  -> MBNMA -0.126 -0.635 0.392  TC_750 vs Placebo_0 0.826  -> direct 0.281 -0.855 1.481  -> indirect 0.147 -0.757 1.058  -> MBNMA 0.193 -0.487 0.884  TC_250 vs Placebo_0 0.813  -> direct 0.118 -0.614 0.824  -> indirect 0.257 -0.725 1.290  -> MBNMA 0.161 -0.408 0.718  MIX_1000 vs Placebo_0 0.524  -> direct -0.020 -0.945 0.917  -> indirect 0.316 -0.116 0.742  -> MBNMA 0.244 -0.138 0.624  MIX_750 vs Placebo_0 0.451  -> direct -0.103 -1.181 1.034  -> indirect 0.282 -0.112 0.698  -> MBNMA 0.239 -0.136 0.613  EG_250 vs Placebo_0 0.918  -> direct 0.330 -0.637 1.327  -> indirect 0.410 -0.527 1.383  -> MBNMA 0.367 -0.309 1.077  AE_500 vs Placebo_0 0.489  -> direct 0.736 0.381 1.108  -> indirect 0.459 0.073 0.883  -> MBNMA 0.610 0.347 0.889  AE_250 vs Placebo_0 0.687  -> direct 0.594 0.046 1.177  -> indirect 0.528 0.252 0.845  -> MBNMA 0.540 0.297 0.813 |
| --- |
|  |
| \| 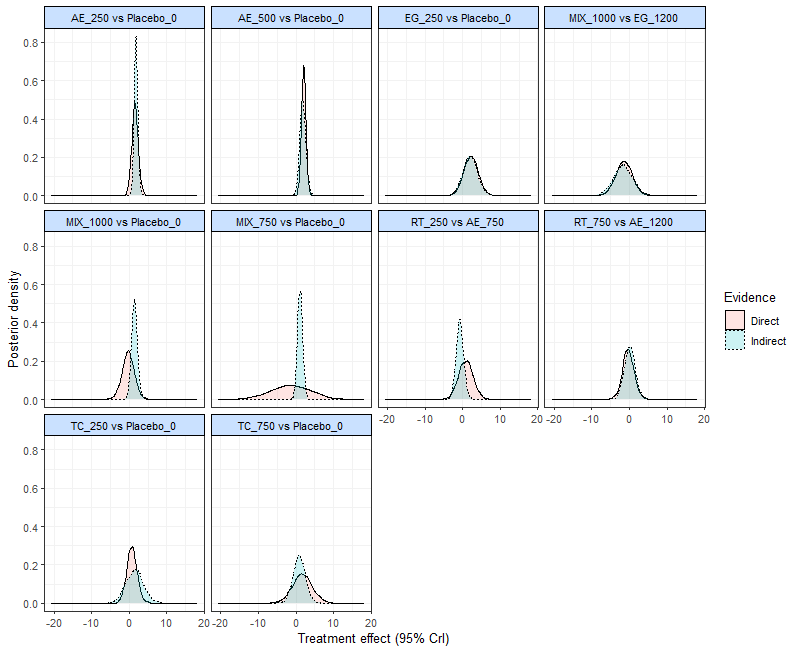 \| \| --- \|   *Appendix Figure 2. Node splitting analysis (density plot)* |

**5.Appendix File 5: Nonlinear functions and models fit comparison**

Meta-analyses (i.e., "split" NMAs) of different doses of physical activity as independent and unrelated treatments were performed. This step helps to determine which function is better suited to the data and is subsequently used in a model-based network meta-analysis (MBNMA). *Appendix Fig. 5 and Appendix Fig. 6* show the different responses of each dose to overall exercise and to different types of exercise.

***
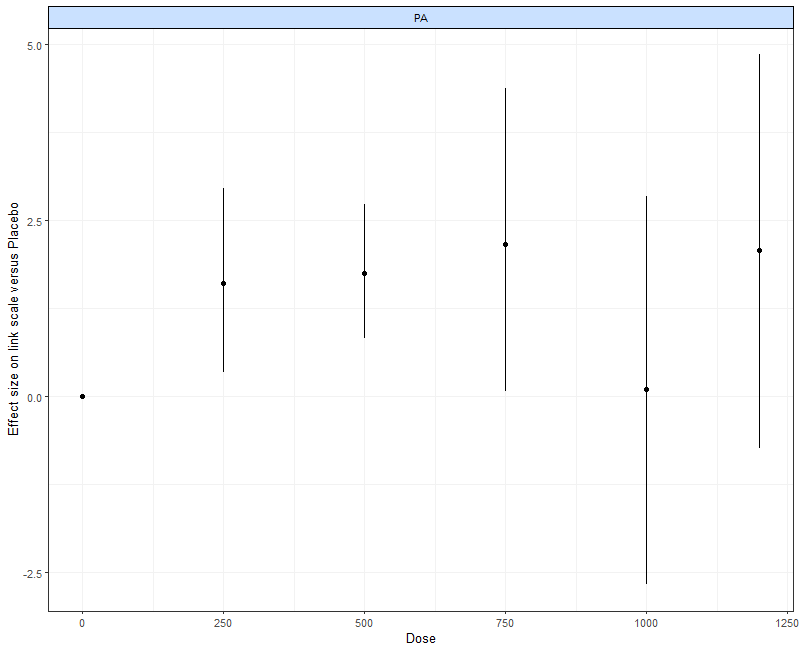
***

***Appendix Figure 3. “Split” NMA of overall exercise***

***
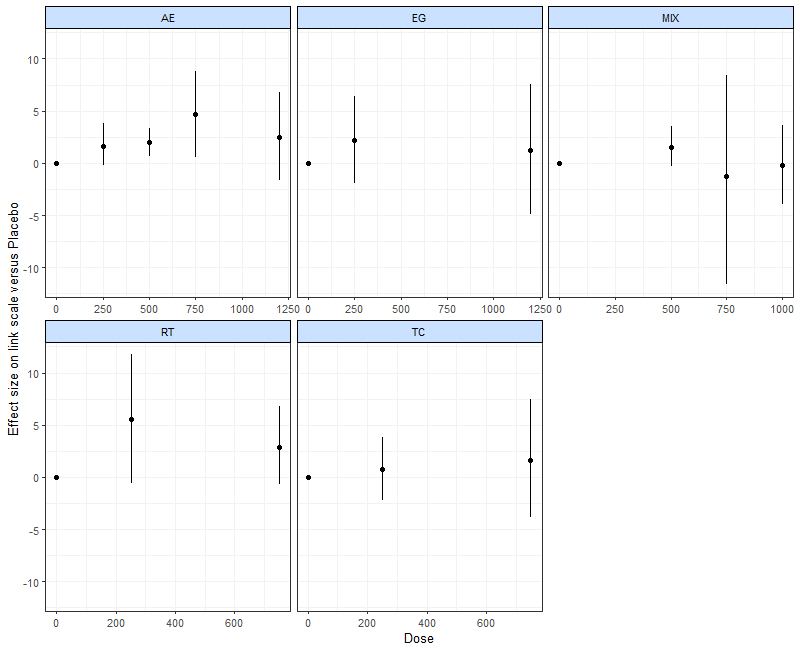
***

***Appendix Figure 4. “Split” NMA of different PA agents***

***Appendix Table 3. Models fit comparison***

| **Model** | **DIC** | **SD** | **Deviance** | **Residual deviance** | **pD** |
| --- | --- | --- | --- | --- | --- |
| Emax（common intervention effects） | 237.3 | NA | 205.730 | 122.743 | 32.1 |
| Emax（random intervention effects） | 192.1 | 0.434 | 141.108 | 58.120 | 49.4 |
| Restricted cubic spline (common intervention effects;3knots) | 242.4 | NA | 205.884 | 122.897 | 37.2 |
| Restricted cubic spline (random intervention effects;3knots) | 190.2 | 0.515 | 140.394 | 57.407 | 51.6 |
| Non-Parameter (common intervention effects) | 245.3 | NA | 212.979 | 129.992 | 33 |
| Non-Parameter (random intervention effects) | 192.2 | 0.594 | 140.237 | 57.249 | 51.1 |
| Exponential (common intervention) | 236.4 | NA | 205.089 | 122.102 | 31.7 |
| Exponential (random intervention) | 192.5 | 0.432 | 140.832 | 57.845 | 49.1 |

***Note. DIC = Deviance Information Criterion; SD = Between study Standard Deviation; pD: Number of estimated parameters; NA = Not Applicable. The SD is presented as the main value and. DIC is an estimate of expected predictive error (lower deviance is better).***

Further to model fit indices, deviance plots showing the contribution of each data point to the residual deviance are also useful to confirm the robustness of model selection. Each data point should contribute about 1 to the posterior mean deviance, which indicates a good model fit. The deviance plot for overall (Appendix Figure 7) and treatment effects (Appendix Figure 8) confirm the robustness of our model selection.

***Appendix Figure 5. Deviance plot at overall PA level***


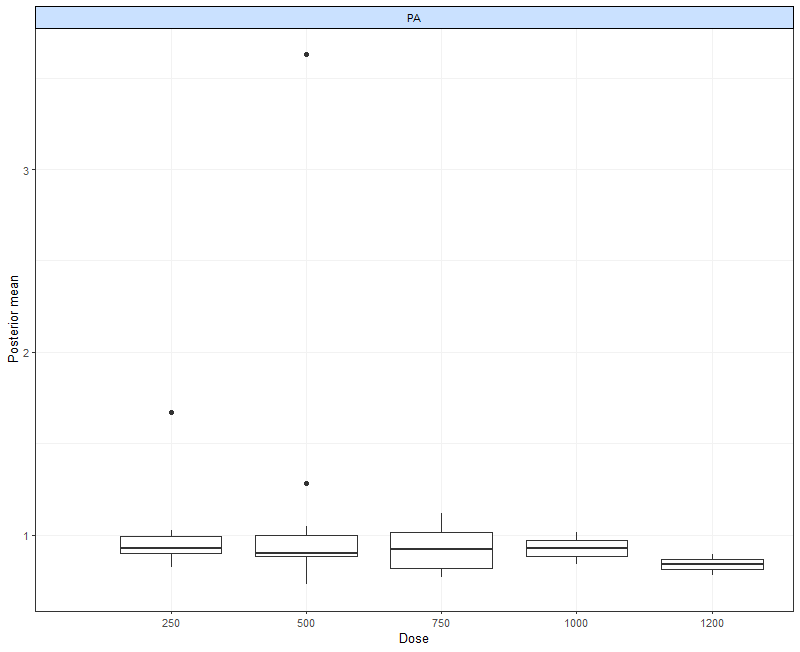


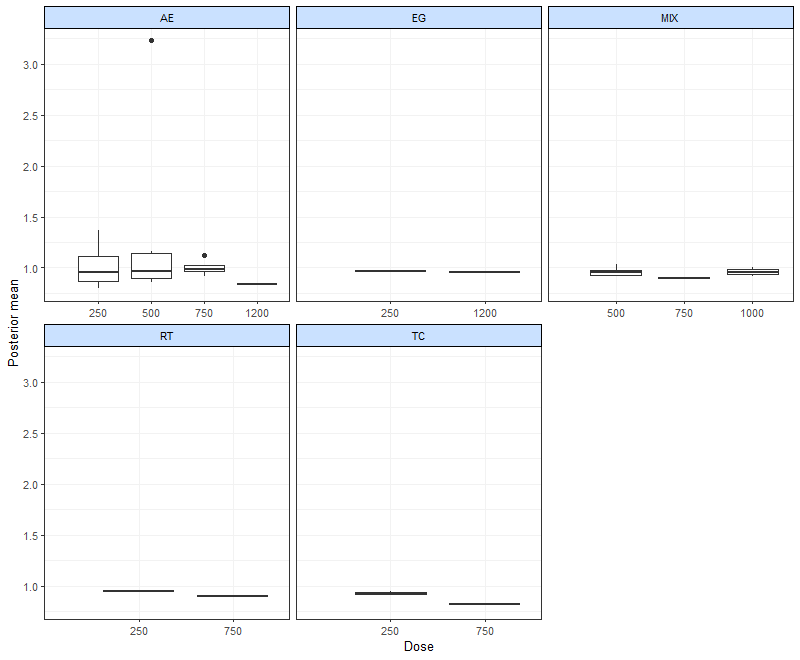


***Appendix Figure 6. Deviance plots at PA level***

**
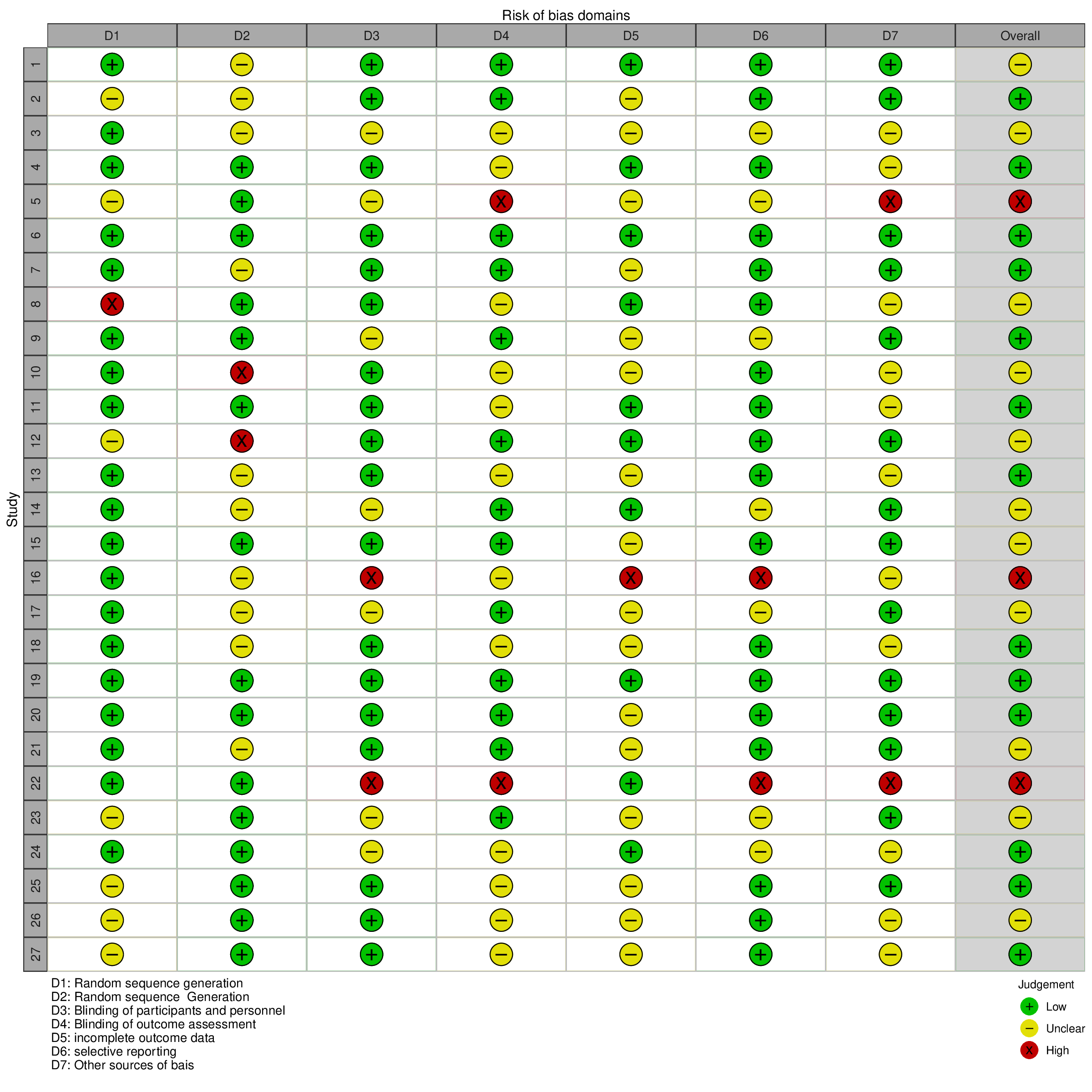
6.**[**Appendix File**](#_heading=h.ihv636) **6**[**:studylevel Risk of Bias analysis**](#_heading=h.ihv636)

[***Appendix Figure 7. Studylevel Risk of Bias analysis***](#_heading=h.41mghml)

**
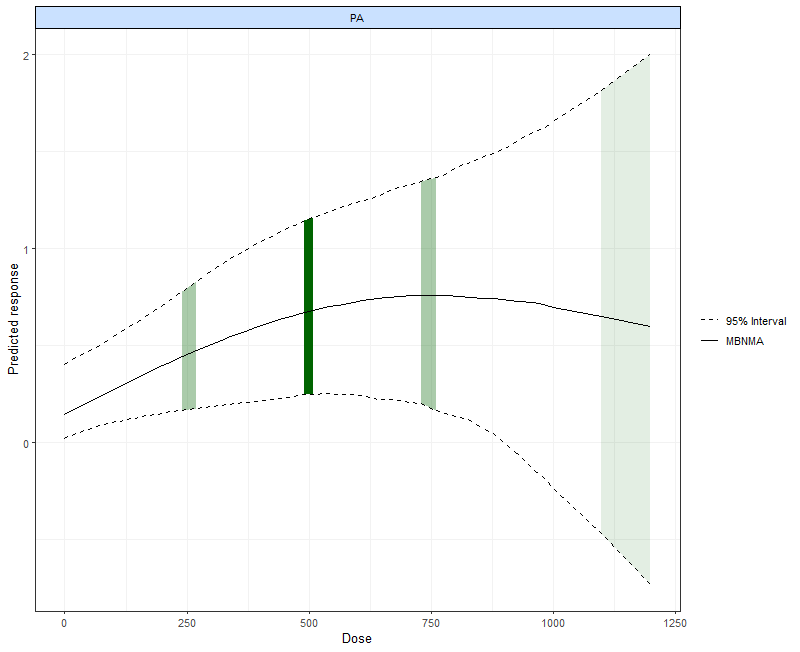
7.[Appendix File](#_heading=h.2grqrue) 7**[**: Sensitivity analysis including only studies with low**](#_heading=h.2grqrue)

[***Appendix Figure 8. Doseresponse curve between exercise and changes in cognitive function after including only studies with low risk of bias***](#_heading=h.vx1227)

**8.Appendix File 8: Definitions of exercise types and non-exercise training control**

| **Abbreviation** | **Full name** | **Definitions** |
| --- | --- | --- |
| AE | Aerobic Exercise | Aerobic exercise is performed by repeating sequences of light-to-moderate intensity activities for extended periods of time. e.g., walking, bicycle, and treadmill training etc. |
| CON | Control group | Non-exercise intervention, usual care, or health education |
| MT | Mixed Exercise Program | Two or more of the above specific types of exercise training (if it is only part of warm-up or relaxation, it is not considered as multi-mode) |
| RT | Resistance Training | Exercise training designed to improve the strength, power, endurance and size of skeletal muscles. |
| TC | Tai Chi | It is an internal Chinese martial art practiced for defense training, health benefits, and meditation. |
| EG | Exargeme | "Exergame" is a combination of the words "exercise" and "game" and refers to video games that incorporate elements of sports. Such games are designed to promote physical activity in an interactive way, allowing players to Get physical exercise while playing games. Exergames can run on a variety of platforms, including computers, game consoles (such as Nintendo Wii, Xbox Kinect, PlayStation Move), and mobile devices, and they achieve this by requiring players to move their body to operate the game. The effects of exercise. |

**9.Appendix File 9: The list of included studies**

1.Lautenschlager, N. T., Cox, K. L., Flicker, L., Foster, J. K., Van Bockxmeer, F. M., Xiao, J., ... & Almeida, O. P. (2008). Effect of physical activity on cognitive function in older adults at risk for Alzheimer disease: a randomized trial. *Jama*, *300*(9), 10271037.

2. Kwak, Y. S., Um, S. Y., Son, T. G., & Kim, D. J. (2007). Effect of regular exercise on senile dementia patients. *International journal of sports medicine*, 471474.

3. Kemoun, G., Thibaud, M., Roumagne, N., Carette, P., Albinet, C., Toussaint, L., ... & Dugué, B. (2010). Effects of a physical training programme on cognitive function and walking efficiency in elderly persons with dementia. *Dementia and geriatric cognitive disorders*, *29*(2), 109114.

4. Tai, S. Y., Hsu, C. L., Huang, S. W., Ma, T. C., Hsieh, W. C., & Yang, Y. H. (2016). Effects of multiple training modalities in patients with Alzheimer’s disease: a pilot study. *Neuropsychiatric Disease and Treatment*, 28432849.

5. Holthoff, V. A., Marschner, K., Scharf, M., Steding, J., Meyer, S., Koch, R., & Donix, M. (2015). Effects of physical activity training in patients with Alzheimer’s dementia: results of a pilot RCT study. *PloS one*, *10*(4), e0121478.

6. Bossers, W. J., van der Woude, L. H., Boersma, F., Hortobágyi, T., Scherder, E. J., & van Heuvelen, M. J. (2015). A 9week aerobic and strength training program improves cognitive and motor function in patients with dementia: a randomized, controlled trial. *The American Journal of Geriatric Psychiatry*, *23*(11), 11061116.

*7.Telenius, E. W., Engedal, K., & Bergland, A. (2015). Effect of a highintensity exercise program on physical function and mental health in nursing home residents with dementia: an assessor blinded randomized controlled trial. PloS one, 10(5), e0126102.*

8. Yang, S. Y., Shan, C. L., Qing, H., Wang, W., Zhu, Y., Yin, M. M., ... & Wu, T. (2015). The effects of aerobic exercise on cognitive function of Alzheimer’s disease patients. *CNS & Neurological DisordersDrug Targets (Formerly Current Drug TargetsCNS & Neurological Disorders)*, *14*(10), 12921297.

9. Cancela, J. M., Ayán, C., Varela, S., & Seijo, M. (2016). Effects of a longterm aerobic exercise intervention on institutionalized patients with dementia. *Journal of science and medicine in sport*, *19*(4), 293298.

10. Hoffmann, K., Sobol, N. A., Frederiksen, K. S., Beyer, N., Vogel, A., Vestergaard, K., ... & Hasselbalch, S. G. (2016). Moderatetohigh intensity physical exercise in patients with Alzheimer’s disease: a randomized controlled trial. *Journal of Alzheimer's Disease*, *50*(2), 443453.

11. Toots, A., Littbrand, H., Boström, G., Hörnsten, C., Holmberg, H., LundinOlsson, L., ... & Rosendahl, E. (2017). Effects of exercise on cognitive function in older people with dementia: a randomized controlled trial. *Journal of Alzheimer's Disease*, *60*(1), 323332.

12. Lamb, S. E., Sheehan, B., Atherton, N., Nichols, V., Collins, H., Mistry, D., ... & Lall, R. (2018). Dementia And Physical Activity (DAPA) trial of moderate to high intensity exercise training for people with dementia: randomised controlled trial. *bmj*, *361*.

13. Venturelli, M., Scarsini, R., & Schena, F. (2011). Sixmonth walking program changes cognitive and ADL performance in patients with Alzheimer. *American Journal of Alzheimer's Disease & Other Dementias®*, *26*(5), 381388.

14. Arcoverde, C., Deslandes, A., Moraes, H., Almeida, C., Araujo, N. B. D., Vasques, P. E., ... & Laks, J. (2014). Treadmill training as an augmentation treatment for Alzheimer’s disease: a pilot randomized controlled study. *Arquivos de neuropsiquiatria*, *72*, 190196.

15. Gaitán, J. M., Boots, E. A., Dougherty, R. J., Oh, J. M., Ma, Y., Edwards, D. F., ... & Okonkwo, O. C. (2019). Brain glucose metabolism, cognition, and cardiorespiratory fitness following exercise training in adults at risk for Alzheimer’s disease. *Brain Plasticity*, *5*(1), 8395.

16. Huang, N., Li, W., Rong, X., Champ, M., Wei, L., Li, M., ... & Lyu, J. (2019). Effects of a modified Tai Chi program on older people with mild dementia: a randomized controlled trial. *Journal of Alzheimer's Disease*, *72*(3), 947956.

17. Nyman, S. R., Ingram, W., Sanders, J., Thomas, P. W., Thomas, S., Vassallo, M., ... & BarradoMartín, Y. (2019). Randomised controlled trial of the effect of Tai Chi on postural balance of people with dementia. *Clinical Interventions in Aging*, 20172029.

18. Sampaio, A., Marques, E. A., Mota, J., & Carvalho, J. (2019). Effects of a multicomponent exercise program in institutionalized elders with Alzheimer’s disease. *Dementia*, *18*(2), 417431.

19. Liu, I. T., Lee, W. J., Lin, S. Y., Chang, S. T., Kao, C. L., & Cheng, Y. Y. (2020). Therapeutic effects of exercise training on elderly patients with dementia: a randomized controlled trial. *Archives of Physical Medicine and Rehabilitation*, *101*(5), 762769.

20. van Santen, J., Dröes, R. M., Twisk, J. W., Henkemans, O. A. B., van Straten, A., & Meiland, F. J. (2020). Effects of exergaming on cognitive and social functioning of people with dementia: a randomized controlled trial. *Journal of the American Medical Directors Association*, *21*(12), 19581967.

21. Liao, Y. Y., Chen, I. H., Hsu, W. C., Tseng, H. Y., & Wang, R. Y. (2021). Effect of exergaming versus combined exercise on cognitive function and brain activation in frail older adults: A randomised controlled trial. *Annals of Physical and Rehabilitation Medicine*, *64*(5), 101492.

22. Yu, F., Salisbury, D., & Mathiason, M. A. (2021). Interindividual differences in the responses to aerobic exercise in Alzheimer's disease: Findings from the FITAD trial. *Journal of Sport and Health Science*, *10*(1), 6572.

23. Yu, F., Vock, D. M., Zhang, L., Salisbury, D., Nelson, N. W., Chow, L. S., ... & Wyman, J. F. (2021). Cognitive effects of aerobic exercise in Alzheimer’s disease: a pilot randomized controlled trial. *Journal of Alzheimer's Disease*, *80*(1), 233244.

24. BorgesMachado, F., Teixeira, L., Carvalho, J., & Ribeiro, O. (2023). Does Multicomponent Physical Exercise Training Work for Dementia? Exploring the Effects on Cognition, Neuropsychiatric Symptoms, and Quality of Life. *Journal of Geriatric Psychiatry and Neurology*, *36*(5), 376385.

25. Vreugdenhil, A., Cannell, J., Davies, A., & Razay, G. (2012). A community‐based exercise programme to improve functional ability in people with Alzheimer’s disease: A randomized controlled trial. *Scandinavian journal of caring sciences*, *26*(1), 1219.

26. Angiolillo, A., Leccese, D., Ciccotelli, S., Di Cesare, G., D'Elia, K., Aurisano, N., ... & Di Costanzo, A. (2023). Effects of Nordic walking in Alzheimer’s disease: A singleblind randomized controlled clinical trial. *Heliyon*, *9*(5).

27. Abbas, R. L., Saab, I. M., AlSharif, H. K., Naja, N., & ElKhatib, A. (2023). Effect of Adding Motorized Cycle Ergometer Over Exercise Training on Balance in Older Adults with Dementia: A Randomized Controlled Trial. *Experimental Aging Research*, *49*(2), 100111.
